# Pulmonary Nontuberculous Mycobacterial Disease in a Tuberculosis-Endemic Setting: Two Cases Illustrating Diagnostic Pitfalls

**DOI:** 10.3390/reports9030265

**Published:** 2026-08-11

**Authors:** Ancuţa-Alina Constantin

**Affiliations:** 1 Department of Cardio-Thoracic Pathology, “Carol Davila” University of Medicine and Pharmacy, 050474 Bucharest, Romania; ancuta-alina.constantin@umfcd.ro; 2 Institute of Pneumology “Marius Nasta”, 050159 Bucharest, Romania

**Keywords:** nontuberculous mycobacteria, pulmonary infections, atypical imaging, typical imaging, real-case management, diagnostic challenges

## Abstract

**Background and Clinical Significance:** Pulmonary disease caused by nontuberculous mycobacteria (NTM) represents an important diagnostic challenge in tuberculosis-endemic settings because its clinical, radiological, and microbiological features may overlap with those of pulmonary tuberculosis (TB). Accurate distinction between these conditions is essential to avoid diagnostic delay and inappropriate treatment. **Case Presentation:** We present two cases that illustrate the heterogeneity of imaging patterns associated with NTM disease. The first case involved a 55-year-old woman with previously treated pulmonary tuberculosis who presented with chronic productive cough, recurrent mild hemoptysis, and progressive nodular-bronchiectatic and cavitary abnormalities. *Mycobacterium avium* was repeatedly isolated from independently collected respiratory specimens and identified using a line probe assay (LPA). The second case involved a 65-year-old man with severe chronic obstructive pulmonary disease (COPD), bronchiectasis, previous tuberculosis, and extensive bilateral fibrocavitary lung disease. Respiratory specimens were acid-fast bacilli-positive, whereas GeneXpert MTB/RIF repeatedly failed to detect the *Mycobacterium tuberculosis* complex. Repeated cultures identified *Mycobacterium xenopi*, including isolates from two sputum specimens and one bronchial aspirate. Treatment was subsequently adapted according to species identification and multidisciplinary assessment. These cases illustrate two major phenotypes of pulmonary NTM disease: nodular-bronchiectatic disease caused by *M. avium* and fibrocavitary disease caused by *M. xenopi*. They emphasize that persistent acid-fast bacilli (AFB) positivity with negative GeneXpert MTB/RIF results should prompt consideration of NTM alongside other differential diagnoses, followed by mycobacterial culture and species-level identification. **Conclusions:** The diagnosis of pulmonary NTM disease requires integration of clinical manifestations, radiological evolution, and repeated microbiological confirmation. Early species identification, multidisciplinary treatment selection, and close follow-up may reduce diagnostic delays and support appropriate individualized management.

## 1. Introduction

Pulmonary infections caused by nontuberculous mycobacteria (NTM) represent a rapidly expanding field within modern pulmonology, increasingly influencing clinical practice through atypical presentations, unpredictable courses, and diagnostic challenges. Based on their growth characteristics in culture, NTM are traditionally classified as rapidly growing mycobacteria, which form mature colonies within 7 days of subculture, and slowly growing mycobacteria, which require more than 7 days. This classification is clinically relevant because it reflects important differences in epidemiology, pathogenicity, antimicrobial susceptibility, and therapeutic management. Clinically relevant rapidly growing NTM include the *Mycobacterium abscessus* complex, *Mycobacterium fortuitum*, and *Mycobacterium chelonae*, whereas the *Mycobacterium avium* complex, *Mycobacterium kansasii*, and *Mycobacterium xenopi* are among the most important slowly growing NTM [1]. Although they may often mimic the clinical and radiological features of pulmonary TB, NTM infections are predominantly acquired from environmental sources [2]. However, person-to-person transmission has been documented in specific settings, particularly for *Mycobacterium abscessus* among patients with cystic fibrosis [3]. NTM pulmonary disease occurs predominantly in patients with chronic lung disease, immunosuppression, or local predisposing factors [4].

NTM comprise more than 190 identified species and subspecies [5], with the most common pathogens implicated in pulmonary infections being the *Mycobacterium avium* complex (MAC) and *Mycobacterium abscessus*, followed by *Mycobacterium xenopi,* and *Mycobacterium kansasii*. These infections occur predominantly in patients with underlying pulmonary risk factors, characterized by structural or functional alterations in the tracheobronchial tree that impair the physiological mucociliary clearance mechanisms [6]. Accurate species identification is crucial, given the distinct antimicrobial resistance profiles and therapeutic responses among different NTM species [5,7].

In this article, we present two clinical cases of pulmonary infection caused by NTM, confirmed through specific microbiological methods, which highlight the clinical heterogeneity, the diversity of species involved, and the diagnostic challenges and therapeutic considerations. The cases include infections with *Mycobacterium avium* and *Mycobacterium xenopi*, illustrating the real-world management of complex clinical scenarios.

## 2. Case Presentation

### 2.1. Case 1

A 55-year-old woman, a former smoker (15 pack-years), with a history of pulmonary tuberculosis (TB) treated in 2007 and 2009 (without bacteriological confirmation), presented with a productive cough, mucopurulent sputum, recurrent mild hemoptysis, and fatigue that had persisted for more than one year. She denied symptoms suggestive of acute respiratory infection.

**Figure 1:**
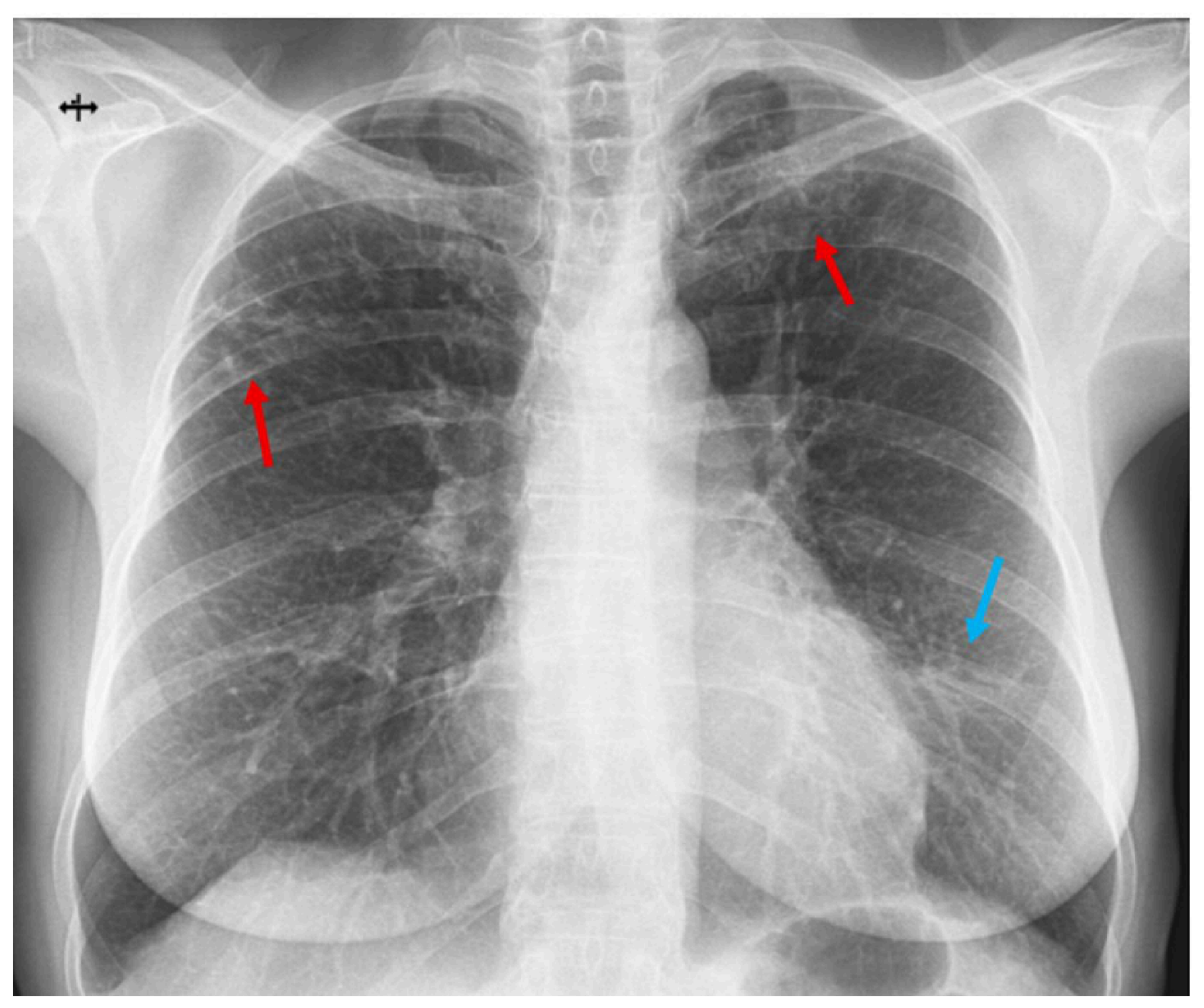
Chest X-ray posteroanterior view showing reticular and reticulonodular opacities of moderate intensity, partially confluent in areas, predominantly distributed in the upper third of both lung fields, more pronounced on the right side (red arrows). A moderate-intensity opacity with irregular margins is also observed adjacent to the cardiac silhouette (blue arrow).

Laboratory tests showed a mild inflammatory syndrome (ESR 49 mm/h) and spirometry demonstrated a mild restrictive ventilatory defect (FVC 74% predicted). Chest radiography revealed bilateral upper-lobe reticulonodular opacities with a left paracardiac infiltrate (Figure 1).

**Figure 2:**
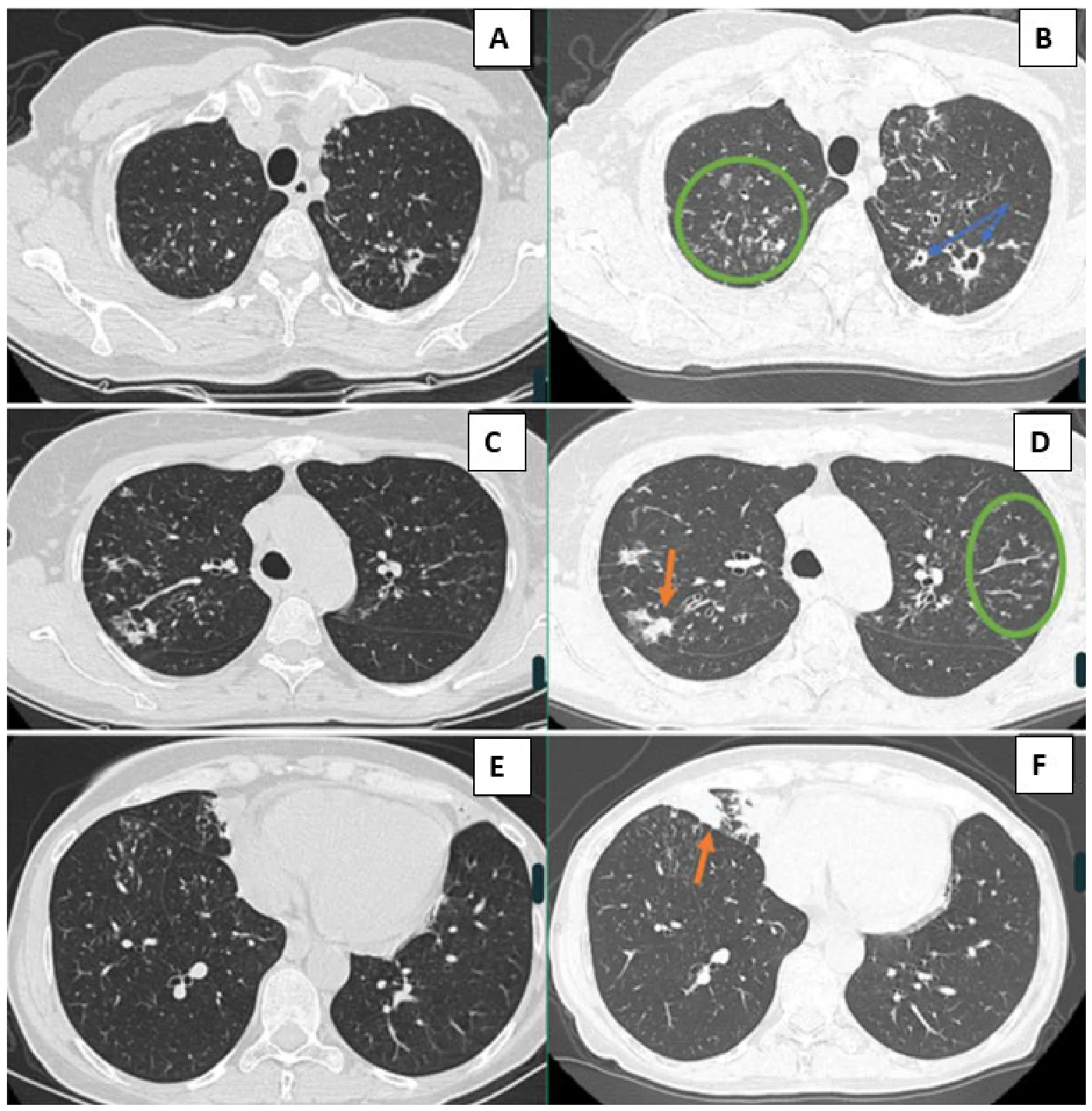
Comparative chest CT images obtained in February 2023 and January 2025, illustrating radiological disease progression at corresponding anatomical levels. (**A**,**C**,**E**) CT images obtained in February 2023; (**B**,**D**,**F**) corresponding CT images obtained in January 2025. (**A**,**B**) Comparative images showing the development of left apical cavitary lesions (blue arrows) and progression of reticulomicronodular infiltrates with a “tree-in-bud” pattern (green circle). (**C**,**D**) Comparative images demonstrating extension of the consolidation areas (orange arrow) and progression of the reticulomicronodular “tree-in-bud” abnormalities (green circle). (**E**,**F**) Comparative images showing further extension of the pulmonary consolidation (orange arrow).

Follow-up chest CT performed in January 2025 demonstrated disease progression, including newly developed left apical cavitary lesions, extension of consolidations, and bilateral reticulonodular infiltrates with a characteristic “tree-in-bud” pattern (Figure 2). Repeat pulmonary function testing showed progression to an obstructive ventilatory defect with significant bronchodilator reversibility, leading to a concomitant diagnosis of asthma.

Given the patient’s previous history of tuberculosis and progressive radiological abnormalities, recurrent pulmonary tuberculosis was initially considered. However, the combination of persistent radiological progression, acid-fast bacilli (AFB) detection in the bronchial aspirate, and repeatedly negative GeneXpert MTB/RIF results prompted consideration of NTM pulmonary disease alongside other differential diagnoses and led to further culture-based and molecular species identification.

During the investigations performed in 2022 and 2023, bronchoscopy with bronchoalveolar lavage (BAL) and bronchial aspiration was undertaken because of persistent respiratory symptoms and radiological abnormalities. Bronchoscopy revealed diffuse bilateral bronchitic changes with mucopurulent secretions. Smear microscopy of the bronchial aspirate was positive for AFB, while mycobacterial culture in liquid medium yielded nontuberculous mycobacteria. Species identification using a line probe assay (LPA) identified *Mycobacterium avium*.

During follow-up, a second independent sputum specimen remained AFB smear-negative, and the GeneXpert MTB/RIF assay did not detect *Mycobacterium tuberculosis* complex. However, mycobacterial culture again yielded *M. avium*, thereby fulfilling the microbiological component of the diagnostic criteria for pulmonary NTM disease. The second isolate subsequently underwent GenoType NTM-DR testing, which confirmed *M. avium* and demonstrated the absence of mutations in the *rrl* (23S rRNA) and *rrs* (16S rRNA) genes associated with macrolide and aminoglycoside resistance, respectively.

At the 2025 reassessment, AFB smear microscopy and GeneXpert MTB/RIF remained negative, and mycobacterial cultures were also negative.

Specific treatment was initially delayed because the patient was repeatedly lost to follow-up despite microbiological confirmation of NTM disease. Following multidisciplinary review by the Romanian National MDR-TB Commission (the Romanian National MDR-TB Commission is a multidisciplinary national expert committee responsible for approving individualized treatment regimens for complex tuberculosis and NTM cases), an individualized regimen consisting of rifampicin, ethambutol, and a macrolide was initiated, with scheduled clinical, microbiological, and radiological follow-up.

Delayed treatment resulted in progressive radiological disease with new cavitary lesions and worsening respiratory symptoms before therapy could be initiated, emphasizing the importance of early diagnosis and patient adherence.

### 2.2. Case 2

A 65-year-old man with a 65 pack-year smoking history, long-term occupational exposure to respiratory irritants, severe chronic obstructive pulmonary disease (COPD) (Global Initiative for Chronic Obstructive Lung Disease (GOLD) IV, Group E), bilateral bronchiectasis, and previously treated pulmonary tuberculosis presented with chronic productive cough, progressive dyspnea (modified Medical Research Council (mMRC) grade 4), weight loss, and fatigue. He had previously completed treatment for *Mycobacterium xenopi* pulmonary disease in August 2022.

**Figure 3:**
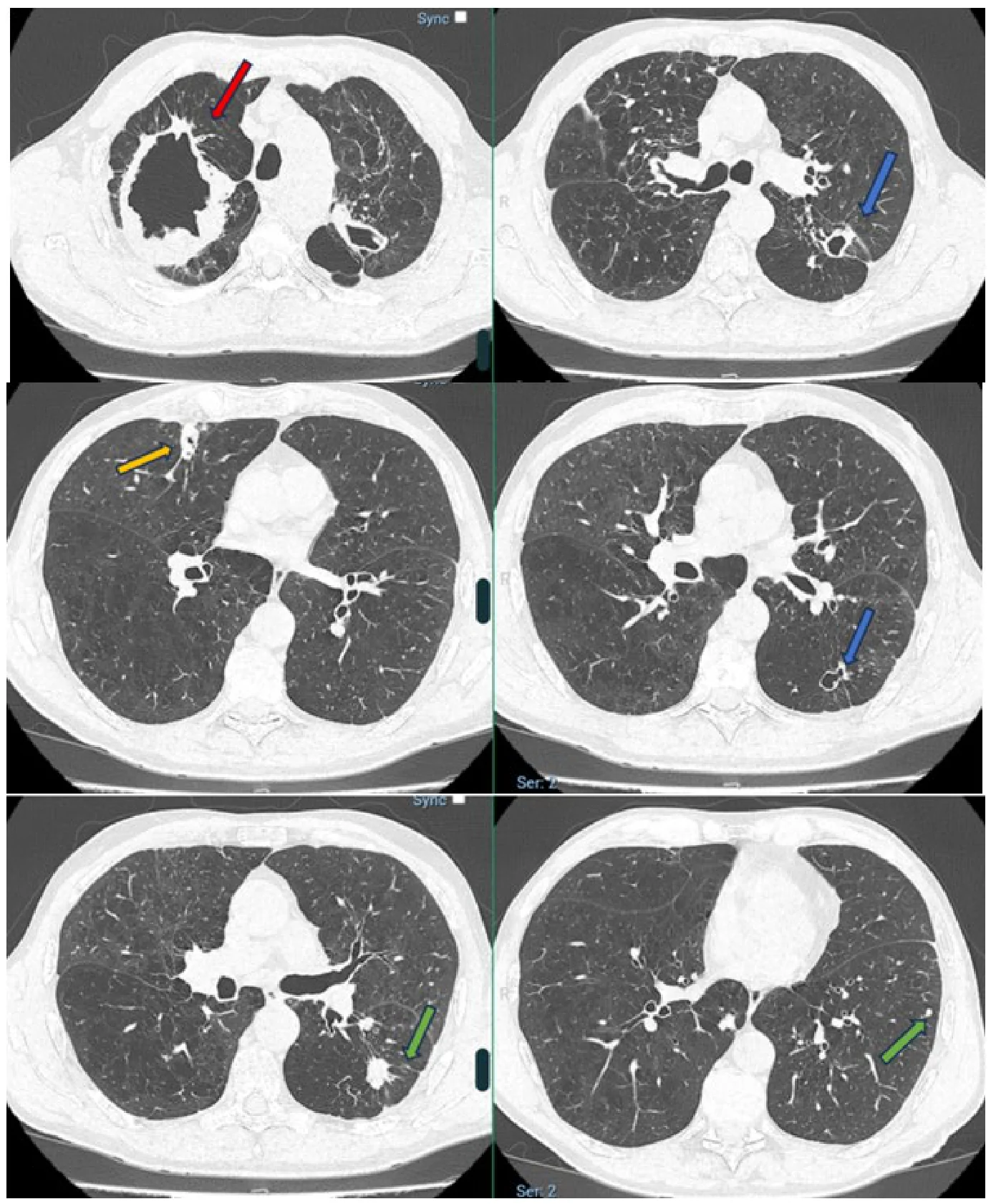
Thoracic CT scan showing a 9.7 × 7.4 cm thick-walled lesion with an irregular internal contour and non–iodine-enhancing areas, containing aerated components and adjacent interstitial thickening, located in the apical and posterior segments of the right upper lobe (red arrow). Inter-connected cavitary lesions are also seen in the apicoposterior and apical segments of the left upper lobe (blue arrows), featuring a draining bronchus and thickened walls, the largest measuring 3 × 2.5 cm. Diffuse pulmonary nodules are present, some calcified and others displaying a “tree-in-bud” pattern, particularly in the left lower lobe (green arrows). A newly developed pseudonodular pulmonary consolidation with adjacent micronodules, slightly iodine-enhancing, is observed in the middle lobe (yellow arrow). The lung parenchyma is markedly emphysematous.

Laboratory evaluation demonstrated a marked inflammatory syndrome (CRP 146 mg/L, ESR 119 mm/h), mild leukocytosis with neutrophilia, normocytic anemia, and transient hepatic cytolysis. Spirometry showed a very severe mixed ventilatory defect (FEV_1_ 32% predicted, FVC 55% predicted). Thoracic CT revealed extensive bilateral cavitary lesions, diffuse bronchiectasis, severe emphysema, and multiple micronodules with a characteristic “tree-in-bud” pattern (Figure 3).

The combination of AFB detected on sputum microscopy, extensive cavitary disease, and a history of tuberculosis initially raised concern for recurrent pulmonary tuberculosis. However, AFB smear positivity together with repeatedly negative GeneXpert MTB/RIF results prompted consideration of NTM pulmonary disease and other alternative diagnoses, pending confirmation by mycobacterial culture and species identification.

Initial sputum examination demonstrated an AFB-positive smear (nine acid-fast bacilli per microscopic field). A repeat sputum specimen was AFB smear-negative, while GeneXpert MTB/RIF testing did not detect *Mycobacterium tuberculosis* complex. However, mycobacterial culture performed on liquid medium yielded nontuberculous mycobacteria. Species identification was subsequently performed by LPA, confirming *Mycobacterium xenopi.* Fungal cultures additionally yielded *Candida* spp. and one colony of filamentous fungi. Considering the patient’s previous history of pulmonary NTM disease, the case was reviewed by the institutional multidisciplinary MDR Committee, which recommended obtaining two concordant positive cultures before reinitiating species-directed treatment, in accordance with the Romanian National Tuberculosis Control Programme recommendations. The laboratory records documented multiple *M. xenopi* isolates over time, including three separate isolates in 2024—comprising two sputum isolates and one bronchial aspirate isolate. The microbiological findings, together with the compatible clinical presentation and radiological progression, supported the diagnosis of pulmonary *M. xenopi* disease.

No sequencing-based confirmation or isolate-specific antimicrobial susceptibility testing results were available for the *M. xenopi* isolates; treatment selection was therefore based on species identification, disease severity, multidisciplinary expert review, and current species-directed recommendations.

Empirical first-line antituberculosis therapy was initiated according to national guidelines while awaiting culture results because of the patient’s severe cavitary disease and high clinical suspicion of tuberculosis. Following microbiological confirmation of *M. xenopi*, treatment was modified after review by the Romanian National MDR-TB Commission to an individualized multidrug regimen comprising a macrolide, fluoroquinolone, rifampicin, ethambutol, and amikacin for at least 12 months after culture conversion. Supportive COPD therapy and treatment of concomitant bacterial and fungal infections were continued.

**Figure 4:**
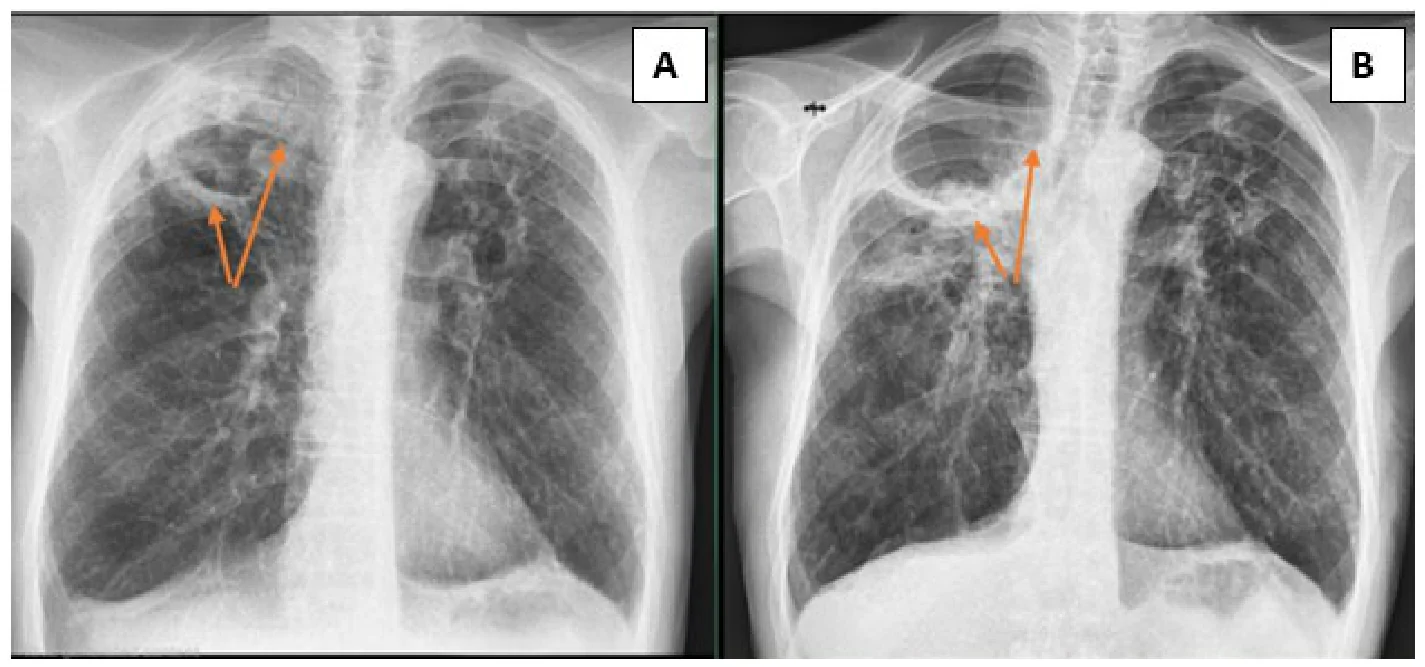
Posteroanterior chest radiographs. (**A**) Initial evaluation, showing the right apical cavitary lesion with intracavitary opacity/content (orange arrows). (**B**) Follow-up radiograph performed four months after treatment initiation, showing a slight reduction in the intracavitary content of the right apical lesion (orange arrows), while the overall radiographic appearance remains largely comparable to the previous examination.

Four months after treatment initiation, chest radiography demonstrated partial regression of the right apical cavitary lesion, while the patient experienced clinical stabilization. Long-term microbiological and radiological follow-up remains ongoing. Follow-up chest radiography, performed four months after treatment initiation during a consultation for an exacerbation of COPD, revealed a slightly favorable evolution of the previously described cavitary lesion, with a mild reduction in the content of the right apical cavity (Figure 4).

## 3. Discussion

Pulmonary nontuberculous mycobacterial disease has become an increasingly recognized clinical entity worldwide, particularly among patients with structural lung disease. However, diagnosis remains challenging in countries where TB is endemic because the clinical manifestations, radiological abnormalities, and even microbiological findings frequently overlap with those of pulmonary TB. Consequently, delayed diagnosis and inappropriate empirical antituberculosis treatment remain common in routine clinical practice [2,3,4,5].

The two cases presented illustrate two distinct phenotypes of NTM pulmonary disease. The first patient developed slowly progressive nodular-bronchiectatic disease caused by *Mycobacterium avium*, whereas the second patient presented with extensive fibrocavitary disease due to *Mycobacterium xenopi* in the setting of severe COPD and previous pulmonary TB [5,8].

The first case is representative of the nodular-bronchiectatic form of *Mycobacterium avium* complex pulmonary disease. This phenotype is characterized by multifocal bronchiectasis, small nodules, and a “tree-in-bud” pattern, reflecting endobronchial spread of infection [8]. Although this presentation has classically been associated with the so-called Lady Windermere syndrome [9], more recent evidence indicates that nodular-bronchiectatic MAC disease occurs in a broader patient population and should not be regarded as a distinct clinical syndrome [4,10]. In our patient, the progressive radiological abnormalities together with repeated isolation of *M. avium* fulfilled the ATS/ERS/ESCMID/IDSA diagnostic criteria for NTM pulmonary disease [5]. Nevertheless, treatment initiation was substantially delayed because recurrent tuberculosis was initially considered more likely and the patient was repeatedly lost to follow-up. This delay resulted in progression from predominantly nodular disease to cavitary involvement, highlighting the potential consequences of postponing appropriate therapy.

The second case illustrates the fibrocavitary form of NTM pulmonary disease, which is frequently associated with *M. xenopi* infection. This phenotype predominantly affects older men with advanced smoking-related lung disease, COPD, emphysema, and previous pulmonary tuberculosis [11,12,13]. In our patient, the diagnosis was supported by concordant clinical, radiological, and microbiological findings. Chronic productive cough, progressive dyspnea, weight loss, and fatigue fulfilled the clinical component, while extensive bilateral cavitary lesions, diffuse bronchiectasis, and multiple nodules with a “tree-in-bud” pattern fulfilled the radiological component. The microbiological criterion was met by repeated isolation of *M. xenopi* from respiratory specimens collected on separate occasions, including three distinct isolates documented in 2024, with species identification performed using LPA. Thus, the findings supported true *M. xenopi* pulmonary disease rather than transient isolation or colonization. Because AFB microscopy cannot distinguish the *M. tuberculosis* complex from NTM, the combination of smear positivity, extensive cavitary destruction, and previous tuberculosis initially justified empirical antituberculosis treatment while culture results were pending. However, repeatedly negative GeneXpert assays and subsequent species identification permitted reassessment of the diagnosis and modification of treatment according to current recommendations [5].

Although underlying structural lung disease may facilitate transient NTM isolation or airway colonization, the isolates in both cases were considered clinically significant because the microbiological findings were reproducible and concordant with active clinical and radiological disease. In Case 1, *M. avium* was recovered from a bronchoscopic respiratory specimen and subsequently from an independent sputum specimen, in the setting of persistent respiratory symptoms and progressive nodular-bronchiectatic and cavitary abnormalities. In Case 2, *M. xenopi* was repeatedly isolated from three separately collected respiratory specimens in 2024, comprising two sputum specimens and one bronchial aspirate, alongside persistent respiratory and constitutional symptoms and progressive fibrocavitary lung disease. Therefore, the isolates were not interpreted as incidental colonization, but as fulfilling the clinical, radiological, and microbiological criteria for pulmonary NTM disease.

Previous pulmonary tuberculosis represented a major predisposing factor in both patients. Structural lung abnormalities resulting from previous TB, including fibrosis, bronchiectasis, and residual cavities, impair mucociliary clearance and create a favorable environment for persistent colonization and infection by environmental mycobacteria. These observations are consistent with previous epidemiological studies demonstrating that a history of pulmonary TB substantially increases the risk of subsequent NTM pulmonary disease [14,15,16,17]. Likewise, the coexistence of severe COPD in the second patient represents another well-established risk factor identified in a recent systematic review and meta-analysis [18].

The management strategies adopted in both cases are consistent with current ATS/ERS/ESCMID/IDSA recommendations, which emphasize that diagnosis should rely on the integration of compatible clinical manifestations, characteristic radiological findings, and repeated microbiological confirmation rather than on microbiological results alone [5]. Species identification is particularly important because treatment regimens, expected therapeutic response, and prognosis differ considerably among NTM species. While MAC pulmonary disease is generally managed with a macrolide-based three-drug regimen, extensive cavitary *M. xenopi* disease frequently requires more intensive multidrug therapy, including an aminoglycoside during the initial phase of treatment [5].

These cases highlight the pivotal role of microbiological investigations in establishing the diagnosis of pulmonary NTM disease. AFB smear microscopy alone cannot differentiate the Mycobacterium tuberculosis complex from nontuberculous mycobacteria [1,5]. Importantly, a negative GeneXpert MTB/RIF result does not itself suggest NTM pulmonary disease. Rather, persistent AFB smear positivity together with repeatedly negative GeneXpert MTB/RIF results should prompt consideration of NTM alongside other differential diagnoses and lead to mycobacterial culture and species-level identification. Molecular identification methods, such as LPA, provide rapid and reliable differentiation of clinically relevant NTM species [1,19]. In tuberculosis-endemic settings, the combined use of smear microscopy, GeneXpert MTB/RIF testing, mycobacterial culture, and molecular species identification is particularly valuable for avoiding diagnostic delays and ensuring appropriate species-directed management [1,5].

Differences in prognosis between NTM species have also been demonstrated in large cohort studies. Wang et al. reported that long-term mortality varied according to infecting species, with poorer outcomes observed among patients infected with *M. kansasii* and *M. xenopi* than among those with MAC disease. Older age, male sex, comorbidities, and infecting species were identified as independent predictors of mortality [20]. These findings are consistent with the clinical severity observed in our second patient, who presented with multiple adverse prognostic factors, including advanced COPD, extensive cavitary disease, and previous pulmonary tuberculosis.

Finally, these cases emphasize that microbiological confirmation alone is insufficient to optimize patient outcomes. The first case demonstrates how loss to follow-up may permit radiological progression despite an established microbiological diagnosis, whereas the second highlights the necessity of balancing early empirical antituberculosis treatment against timely reassessment once molecular and culture results become available. In both situations, multidisciplinary management and repeated clinical, radiological, and microbiological assessment were essential for establishing the correct diagnosis and selecting individualized treatment [5].

The two cases presented illustrate several practical aspects of the diagnosis and management of pulmonary NTM disease in a tuberculosis-endemic setting:

- Persistent respiratory symptoms associated with positive AFB smears and negative GeneXpert results should prompt early investigation for NTM pulmonary disease, particularly in patients with underlying structural lung abnormalities.
- A previous history of pulmonary tuberculosis is an important predisposing factor for NTM infection, as post-tuberculosis structural lung damage, including bronchiectasis and cavitary lesions, creates a favorable environment for NTM colonization and disease.
- Accurate species identification using molecular methods is essential, because treatment regimens, antimicrobial susceptibility, and prognosis differ substantially among NTM species. Empirical antituberculosis treatment should be reassessed once *Mycobacterium tuberculosis* has been excluded.
- Delayed diagnosis or interruption of follow-up may result in progressive structural lung damage, as illustrated by the development of new cavitary lesions and radiological progression in the patient with *Mycobacterium avium* infection.
- Close follow-up throughout treatment is essential because NTM pulmonary disease usually requires prolonged multidrug therapy. Regular clinical, microbiological, and radiological reassessment allows for evaluation of treatment adherence and response, early detection of drug-related adverse events, and timely adjustment of the therapeutic regimen when necessary.
- Management of pulmonary NTM disease requires integration of clinical, radiological, and microbiological findings, together with multidisciplinary decision-making, to ensure timely initiation of appropriate therapy and long-term monitoring.

## 4. Conclusions

Pulmonary NTM disease should be considered among the differential diagnoses in patients with chronic respiratory symptoms, progressive radiological abnormalities, and AFB-positive respiratory specimens in which GeneXpert MTB/RIF repeatedly fails to detect the *Mycobacterium tuberculosis* complex. These two cases illustrate that integrating radiological evolution, repeated microbiological testing, and species identification is essential for timely diagnosis and individualized therapy, particularly in tuberculosis-endemic regions.

Increased clinician awareness may reduce unnecessary antituberculosis treatment and improve outcomes through earlier diagnosis and species-directed therapy.

## Data Availability

The original data presented in this study are included in this article; further inquiries can be directed to the corresponding author.
